# A Dynamic Risk Appraisal Model and Its Application in VTS Based on a Cellular Automata Simulation Prediction

**DOI:** 10.3390/s21144741

**Published:** 2021-07-11

**Authors:** Yongfeng Suo, Zhihong Sun, Christophe Claramunt, Shenhua Yang, Zhibing Zhang

**Affiliations:** 1 Navigation College, Jimei University, Xiamen 361021, China; yfsuo@jmu.edu.cn (Y.S.); 199861000013@jmu.edu.cn (S.Y.); a415233257@163.com (Z.Z.); 2 Department of Navigation, China Coast Guard Academy, Ningbo 315801, China; 3 Naval Academy Research Institute, BP 600, 29240 Brest Naval, France; christophe.claramunt@ecole-navale.fr

**Keywords:** cellular automata, risk appraisal, traffic simulation, traffic prediction, VTS

## Abstract

The successful implementation of Vessel Traffic Services (VTS) relies heavily on human decisions. With the increasing development of maritime traffic, there is an urgent need to provide a sound support for dynamic risk appraisals and decision support. This research introduces a cellular automata (CA) simulation-based modelling approach the objective of which is to analyze and evaluate real-time maritime traffic risks in port environments. The first component is the design of a CA model to monitor ships’ behavior and maritime fairway traffic. The second component is the refinement of the modelling approach by combining a cloud model with expert knowledge. The third component establishes a risk assessment model based on a fuzzy comprehensive evaluation. A typical scenario was experimentally implemented to validate the model’s efficiency and operationality.

## 1. Introduction

Currently, with the increasing development of the maritime economy favored by globalization, port water vessels tend to be large-scale, have relatively high-speeds, and are intensive, which undoubtedly brings great pressure to a port VTS monitoring center. VTS is recognized internationally as a navigational safety measure through the International Convention on the Safety of Life at Sea 74/78 (SOLAS) [1]. A VTS is based on a multi-technology fusion system that integrates radar, internet, radio communications, AIS (Automatic Identification System), CCTV (Closed-Circuit Television), and other technologies. It provides a complete set of ship traffic service systems used by many maritime departments to control and supervise ships. A VTS system has the capability to monitor ship dynamics and provide information, advice, or instructions to ships, and can also effectively control traffic flow and minimize the risk of ship traffic accidents. The VTS has recently played an important role in traffic safety at sea although operators are often exhausted from heavy work and long periods watching real-time situations. Over the past few years, many novel systems and approaches supporting decision-making processes in VTS centers have been introduced as part of integrated support systems [2,3]. Over the past few decades, research on the impact of changes in technology and navigation aids on maritime work practices has been ongoing [4,5,6,7,8,9,10]. The results of several projects of the European Union (EU) have specifically discussed various aspects of the e-Navigation concept, the proposed solutions, (i.e., MarNIS (2004-2008), ACCSEAS (2007-13), (MONALISA (2010-EU-21109-S), MONALISA 2.0 (2012-EU-21007-S), EfficienSea2 (H2020-EU. 3.4), STM (2014-EU-TM-0206-S)), and the potential impact on the technical systems of shipping society. However, researchers still need to develop real-time information and decision-aided systems to assess the risks associated with a single ship and the risks in relation to other ships acting in a specific sea area. This will make a significant contribution to improving the safety and decision-making of VTS operators and thus to the work of VTS operators. A risk assessment system that provides dynamic risk information and is constantly updated can improve the awareness of developments during workload peaks at Vessel Traffic Service (VTS) centers [11].

The objective of the research presented in this paper was to develop a dynamic risk appraisal system for VTS based on a CA simulation prediction mechanism. The whole approach is based on a ship’s behavior in real-time and fairway traffic that constitute the input of the CA to construct a real-time risk index system for port waters. The CA supports the derivation of a channel prediction model at a lower level of resolution, as well as a definition of the behavioral rules that reflect a ship’s motion in the channel. Risk factors are weighted by combining the CA with an expert-based questionnaire. A combination of cloud models and expert scores is used to determine the weight of each risk indicator. Finally, the risk assessment model is enriched by a fuzzy comprehensive assessment.

The rest of the paper is organized as follows. Section Two briefly introduces related work while Section Three develops the principles behind the CA-based traffic simulation. Section Four describes the risk weighting, the appraisal approach and the experimental evaluation, while Section Five concludes the paper and draws some perspectives for further work.

## 2. Related Work

The development of a successful and ‘intelligent’ VTS is still a challenging task. Besides the common E-Navigation projects that are most often oriented towards information integration and exchange rather than real decision-making systems, there is a novel trend which progressively searches for reliable and operational solutions oriented to track prediction and dynamic risk assessment.

### 2.1. Dynamic Risk Management

Dynamic risk management can be interpreted as identifying and analyzing events that may have an impact on individuals or the environment, assessing the consequences and communicating them to decision-makers [12]. A risk can be roughly evaluated by an analysis of the impact of an accident, which should denote the expected accumulation of negative consequences of a particular activity in a predetermined area and period. The main objective of the VTS monitoring center is to provide real-time forecasts of risks (probabilities and consequences) for the target sea area. Such an envisaged regime should implement the following evaluations [13,14,15]:

(1) Calculate and estimate the probabilities of individual ship collisions, groundings, and near-contact situations dynamically and gain a comprehensive understanding of ship traffic situations;
(2) Estimate the consequences of possible accidents based on the characteristics of the vessel and the sea area;
(3) Provide automatic warnings and guidance to VTS operators for risk assessments in real-time and at sea. VTS can be viewed as a decision-making process related to the implementation of maritime safety measures to mitigate risks. Therefore, VTS operators should provide optimal navigation advice and guidance to vessels in and out of the surrounding waters through VHF communications.

However, a sound integration of the above objectives is not a straightforward task. Although ships currently rely on many automated sensors and automated systems that should favor navigation processes and decisions, the final decision are fully controlled by humans and are therefore still error-prone. Similarly, VTS decisions are also made by humans, and this explains the complexity of maritime risk assessment and decision-making. As the number of ships at sea increases, VTS operators are under high pressure and this generates a high workload and then an increase in traffic risks at sea [16,17]. In particular, it has been observed that the increasing complexity of local maritime traffic conditions can lead to many situations of near misses and near collisions at sea [15]. In a related work, a model of possible conflicts at low resolution was suggested based on AIS data and was applied to the Dutch part of the North Sea [18]. It was observed that worse situations happened when multiple ships converged, and it was recommended that such a situation should be monitored at lower spatial and temporal resolutions. The need for the development of low-level dynamic traffic management has been suggested as a required development as such situations cannot be resolved at the individual ship level as there is indeed a need for a global view of a given situation, real-time information, and decision-making assistance to help VTS operators [19]. Van Westrenen et al. used the concept of complexity to analyze traffic. In the research, it was assumed that a certain threshold in the complexity beyond which the system, in relation to a set of ships within a certain navigable area, may no longer be able to resolve the conflict itself using current regulations, technology, and expertise. The analysis of three actual collisions on the high seas showed that situations of high complexity, which decrease human reliability, can be predicted well in advance, allowing for a safe resolution [20]. Based on the above-mentioned theories about VTS and shore-based services, there have been many studies that have discussed the future research directions of VTS [21,22]. The Sea Traffic Management (STM) Validation Project is a European-based initiative with ambitions to improve maritime safety and efficiency through information sharing in real time. Katie Aylward et al. discussed the role of STM service integration in changing the role of VTS operators in traffic conditions and the potential impact of STM service individuals on VTS operations from the perspective of social technology systems [22].

Two recently developed dynamic risk management projects are IWRIS [23] and BaSSy [24]. IWRIS is a developed technology based on real-time Automatic Identification System (AIS) data for vessels to support the operative decision-making of VTS operators. The core of this method is a data model, which takes real-time AIS data as the input and provides risk estimates to remind VTS operators of possible dangers in a given monitored traffic area. The concept of a computer-based decision support system is implemented for the VTS operator in the BaSSy project, which includes the detection of abnormal vessel conditions that could lead to collision and grounding. The system uses real-time AIS data, and the method is mainly based on geometric data, without considering additional statistics. Decision support tools can predict the movement of the ship, and based on the prediction results, give warnings of possible close encounter situations. This decision support system can perceive and identify abnormal traffic behaviors in advance. Despite their interest, these two approaches still focus more on ship collisions or grounding alarms themselves rather than a systematic evaluation of traffic safety at large.

### 2.2. Track Prediction

Track prediction is an issue of research significance because it plays a vital role in the basic traffic management and decision-making processes. So far, various traffic prediction methods have been proposed. The main algorithms developed for traffic prediction can be divided into three groups: point-based, which only predicts the next step and can only be used for small-sized ships; trajectory-based orbit prediction; and simulation-based algorithms [25].

- In points-based algorithms, the target maritime area is divided into different nonoverlapping two-dimensional cells. When given the current state of the ship, this type of algorithm determines the probability that the ship will occupy other nearby units after a given short time. The algorithms estimate the probability of the next state based on historical data (i.e., training set). This type of algorithm is usually used to predict the position of vessels on a small scale [26,27,28,29].
- Trajectory-based algorithms cluster historical data to extract and classify maritime routes. These routes are modeled, and waypoints are optionally considered for different purposes. Waypoints include harbors, offshore platforms, entries, and exit points in the area [30,31,32].
- Simulation-based algorithms are based on either cellular automata [33,34] or intelligent agents [35,36]. Each ship can be regarded as a definite particle, and the interaction between these particles is determined by the interaction of the vessels’ motions. Thus, the cellular automata approach appears to have a better renewal performance, which may be critical in many maritime contexts, especially in areas with high computational loads.

As far as marine traffic prediction is concerned, ships may impact each other while navigating, and the obliged ship should give way to the priority ship according to the International Regulations for Preventing Collisions at Sea 1972 (COLREGs) [37]. This process is nonlinear and complicated. The point-based algorithms and the trajectory-based algorithms do not take into consideration ship interactions. Due to the high number of iterations of computation, it appears that cellular automata methods, which are more efficient computationally, provide better implementation perspectives than the agent approach.

**Figure 1:**
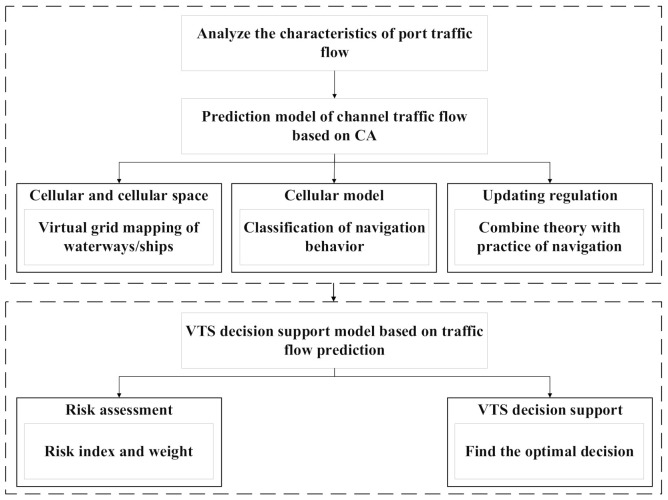
Overall method diagram.

Considering maritime navigation risks from a global perspective can more effectively assist VTS operators in proper navigation management. The objective of this paper is to develop a systematic dynamic risk model based on an integration of different safety factors, which could enhance the accuracy of the system risk appraisal and management. This paper introduces a cellular automata (CA) and simulation approach supported by the Python development framework that first integrates real input data for traffic flow simulation, and secondly brings the simulation results into a risk assessment model for a dynamic risk assessment. The main computational component of the proposed method is the traffic flow simulation based on CA, while the risk assessment is a multicriteria evaluation based on the simulation results. Because the number of ships in the water area is limited, under the framework of the CA algorithm, ships only need to calculate the distance from the ships in and before their own channel and the ships before and after their adjacent channel. The computational efficiency of the proposed method can be used in practical maritime and traffic applications. The overall working idea of the paper is shown in Figure 1.

## 3. CA-Based Traffic Simulation

Simulating traffic in restricted sea areas is not an easy task. All traffic components including ships, the maritime environment, and sailors make up a nonlinear complex system. Any of the ship’s behaviors may generate a change of status and impact, and this potentially generates an unsafe situation and possibly major incidents. Moreover, such traffic updates might be unavoidable, such as a vessel anchored asking for heaving up and proceeding to its berth. Therefore, VTS operators should anticipate the risks that might arise from a behavior change and should be in the position to decide if that request should be approved or not. This leads us to introduce the principles behind a hybrid simulation system model, the objective of which is to simulate the behavior of a traffic system. The whole approach is based on a CA design and update regulations.

The cellular automata algorithm is a dynamic and discrete system controlled by straight local rules. Since cellular automata can simulate the behavior of a complex dynamic system with relatively simple local update rules, the cellular automata theory has been widely applied to the simulation of complex dynamic systems, including traffic systems.

### 3.1. Fairway and Sensitive Area Modeling

**Figure 2:**
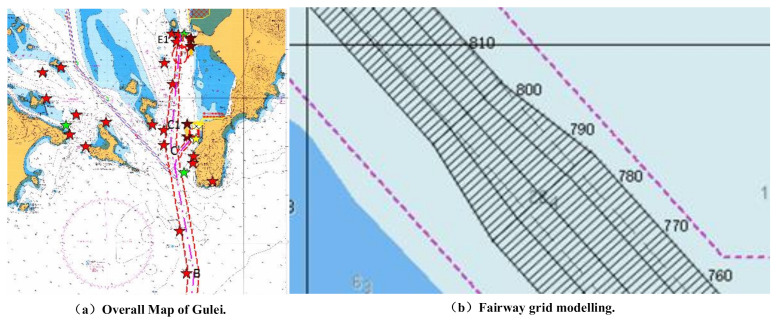
Channel simulation.

As a regular square grid is not always the most appropriate, especially as a given channel is very unlikely to fit such a regular area, a context-based spatial structure was retained and based on the main channel axis as illustrated in Figure 2b. Figure 2a is the overall waterway map of Gulei Port in Zhangzhou, in which the red is the main waterway of Gulei Port. The quadrangle longitude and latitude coordinates of each small grid were generated according to the common ship footprint; this is appropriate for the modelling approach as well as being convenient for ship visualization. Considering that small fishing boats at sea are about 7 m long, the length of the grid along the channel axis was set as 5 m for computational and accuracy purposes.

### 3.2. Cellular Model

In order to reflect the behavior of ship traffic in a port and channel, the cellular-oriented ship model was initiated by two formulas as follows.

(1)→A normal navigating ship and on overtaking ship

**Figure 3:**
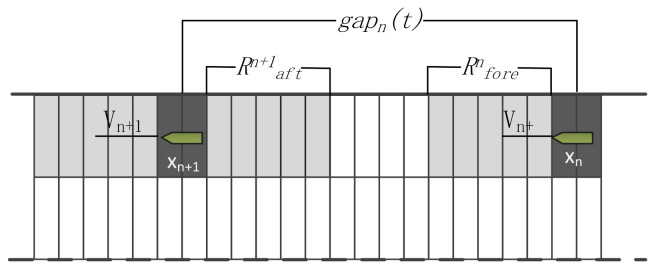
Ship domain cellular.

We applied the navigation principles of the Fujii model, which is considered as a reference for navigation modelling [38], with a narrow waterway of 6 L long and 1.6 L width. The Fujii model was processed as a regular rectangle for modeling convenience, then the ship domain model was defined as follows:

(1) $$R_{fore}=R_{aft}=3\times [\frac{L_{s}}{L_{c}}]$$

where *R_fore_* is the long axis of the front ship, *R_aft_* is the long axis of the bound of the ship, and *L_s_* and *L_w_* denote the ship’s length and width, respectively. *L_c_* and *B_c_* are the cellule’s length and width, respectively (Figure 3).

(2)→The domain of crossing ships 

In common navigation, a ship should try to keep the true course perpendicular to the channel as soon as possible according to the International Regulations for Preventing Collisions at Sea 1972 (COLREGs). Without loss of generality, we assumed that a ship will cross the whole channel vertically, which will have an impact on ships entering and leaving the port within the channel. Accordingly, the ship domain was also treated as a regular rectangle. For the short axis crossing the domain, this model set it as four cells, which are equal to the channel width. The major and minor axes crossing the ship’s neighborhood were derived as follows:

(2) $$R_{cro}=[\frac{\frac{W}{V_{cro}}\times V_{ave}+D_{saf}+B_{cro}}{L_{c}}]$$

where *R_cro_* is the long axis of the crossing ship domain and *W* is the fairway width. This model defines this as four cells, which is the width of the channel. *V_cro_* is the crossing ship’s speed, *V_ave_* is the average speed of the traffic in the fairway, *D_saf_* is the length of ship domain in the traffic lane, *B_cro_* is the width of the crossing ship domain and Lc is the cellule length.

### 3.3. Updating Regulation

This modelling approach is based on the assumption that no congestion is yielded in the maritime area. A regular traffic flow of ships in the channel can be evaluated by the following scenarios:

(1) Free navigation: a current ship is the first ship or the distance between the current ship and the ship in front is greater than the safe distance, which is not affected by the driving status of the ship in front and maintains a constant speed according to its maximum speed. When the initial state is lower than the maximum speed, it accelerates to the maximum speed [39].
(2) Following navigation: the following navigation includes an active and a passive following. Active following means that the current driving status can meet the demand of the ship driving, and the ship is willing to follow the ship ahead. Passive following means that the current driving cannot meet the demand (such as the speed of the forward ship is too slow, or does not meet the economic speed, etc.), overtaking safety conditions are also not satisfied (such as blocking in the opposite direction of the traffic lane, the distance between the current ship and the forward ship is too short, etc.), and the ship can only be forced to slow down to follow the forward ship and sail at the previous speed.

1)→Free Navigation Rules

Free navigation means that the distance between the current ship and the fore ship is greater than the safe distance. This is not affected by the running status of the fore ship and maintains a constant speed according to its normal speed. When the initial status is lower than the normal speed, it accelerates to the normal speed.

(3) $$\left\{\begin{array}{c} \begin{array}{cc} v_{n}(t+1)=\min(v_{n}(t)+1,v_{\max}) & if:gap_{n}(t)<d_{saf}^{n}(t) \end{array}\\ gap_{n}(t)=x_{n+1}(t)-x_{n}(t)-0.5(L^{n}+L^{n+1})+(v_{n+1}-v_{n})\\ d_{saf}^{n}(t)=R_{fore}^{n}+R_{aft}^{n+1} \end{array}\right.$$

where $gap_{n}\left(t\right)$ is the gap between the current ship and the fore ship taking into consideration the so-called membership degree and the speed difference, $d_{saf}^{n}\left(t\right)$ denotes the safe ship domain,$\;L^{n}$ and $L^{n+1}$ are the number of cells occupied by the current ship n and the forward ship n + 1, respectively.

This formula is a mathematical model that describes the iterative relationship between the ship’s speed at the next moment and the ship’s speed at the current moment. In this discrete power system, the speed of the own ship at the next moment is determined by the current relative position and the relationship between the current own ship and the front and rear ships, the captains of the own ship and the front and rear ships, and the speed of the own ship and the front and rear ships.

2)→Following Navigation Rules

As shown in Formula (4), ∆v is the speed difference between the own ship and the previous ship, *r* is the difference between the distance between two adjacent ships and the safe distance at time t. If ∆v>r, the ship should decrease its speed in an emergency until the speed is not higher than the ship ahead, or should increase the speed to overtake. If ∆v<r, the ship will decrease its speed gradually. However, if ∆v<0, the ship’s speed is less than the ship ahead; therefore, the ship’s speed could be increased gradually by the free navigation rules. P is a constant greater than 1. The specific size will be discussed in the follow-up research.

(4) $$\left\{\begin{array}{c} \begin{array}{cc} v_{n}(t+1)=v_{n+1}(t) & if:1<\frac{v_{n}(t)}{v_{n+1}(t)}\leq P \end{array}\\ \begin{array}{c} \begin{array}{c} \begin{array}{cc} v_{n}(t+1)=\min(v_{n}(t)+1,v_{\max}) & if:\Delta v<r \end{array}\\ r=gap_{n}(t)-d_{saf}^{n}(t) \end{array}\\ \Delta v=v_{n}(t)-v_{n+1}(t) \end{array}\\ 1<\frac{v_{n}(t)}{v_{n+1}(t)}\leq P \end{array}\right.$$

When the ship is close enough to the previous ship, the ship can no longer follow the rules of free navigation. In this case, it is necessary to add variables to judge the positional relationship between the own ship and the preceding ship, and the navigation state in discrete time segments to avoid accidents. The two variables *r* and P were added here to refine the judgment standard of the relative relationship between the two ships, making the follow-up rules more stringent.

3)→Overtaking Regulation

**Figure 4:**
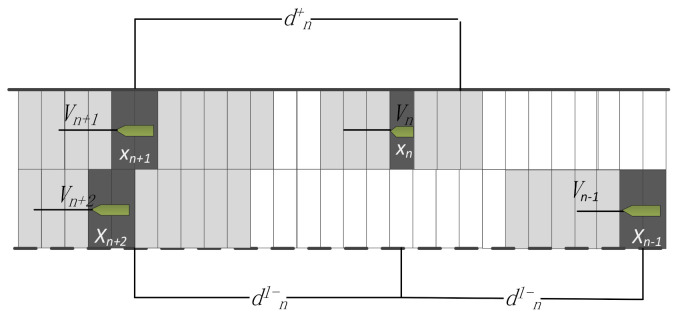
Key parameters to consider when overtaking.

A two-way fairway includes two traffic lanes. The right channel is the default channel, and the left is the overtaking channel. After overtaking, the ship enters the left channel, but the retrieval update keeps pace with the right channel. Therefore, if the pilot has the intention to chase, in addition to maintaining a safe transverse distance, they should also reasonably judge whether the channel meets the conditions for overtaking and try to reduce the ship’s parallel timing. As long as the following overtaking conditions are met, the overtaking can be carried out and the situation of overtaking can be recorded. The following overtaking rules were estimated for the convenience of the simulation. The following three conditions should be satisfied, and they were established not only for the overtaking procedure but also for the return back to the original traffic lane (Figure 4).

(1) The safe distance range between the overtaking ship and the overtaken ship that is about to enter the front ship;
(2) The speed of the rear ship is greater than that of the front ship;
(3) No other ships are running in parallel.

If these three points are satisfied, overtaking can be carried out. The model for alternating traffic lane overtaking is expressed as Formula (5). After overtaking has finished, Formula (6) is used to return to the original traffic lane. The relevant variables are illustrated in Figure 4.

(5) $$\left\{\begin{array}{c} \begin{array}{cc} v_{n}(t+1)=v_{n+1}(t) & if:1<\frac{v_{n}(t)}{v_{n+1}(t)}\leq P \end{array}\\ \begin{array}{c} \begin{array}{c} \begin{array}{cc} v_{n}(t+1)=\min(v_{n}(t)+1,v_{\max}) & if:\Delta v<r \end{array}\\ r=gap_{n}(t)-d_{saf}^{n}(t) \end{array}\\ \Delta v=v_{n}(t)-v_{n+1}(t) \end{array}\\ 1<\frac{v_{n}(t)}{v_{n+1}(t)}\leq P \end{array}\right.$$

(6) $$\left\{\begin{array}{c} d_{n}^{l-}(t)\geq d_{saf}^{n-1}(t)\geq v_{\max}\\ d_{n}^{l+}\geq d_{saf}^{n}(t) \end{array}\right.$$

where $d_{n}^{ly}\left(t\right)$ is the number of cells between the current ship and the ‘after’ ship, *l* denotes the other traffic lane (if the vessel is under overtaking, *l* depicts the left traffic lane). If it has finished overtaking, *l* is the right traffic lane), *y* could be set to + or −, the former means the ‘fore’ ship, the latter is the ‘after’ ship.

We considered ships navigating in the fairway. The crossing ships and obstructing ships do not perform the update rules, these rules only impact ships in the fairway. The neighbor ranges of the above two kinds of ship and the overtaking rules of acceleration will record a crossing or obstruction encounter; therefore, the cellular attribute variables will be updated accordingly. In the simulation system, the regional rule of fairway priority was formulated, that is, all the crossing ships will avoid the direct ships in the fairway. When the ship comes to the cell where the interleaving region of the branch channel and this channel is located, if there is an opposite incoming ship in the interleaving region, the cell can record the encounter situation of the first interleaving region and attribute variables.

## 4. Risk Weight and Appraisal

It is widely known that stillness is a relative concept while motion is a much more absolute notion, so the establishment of a risk index system should be able to dynamically, timely, and accurately reflect the navigation status of a given ship. However, in existing early warning indicators, there is a lack of a dynamic tracking of ships and ship traffic flow parameters, such as ship speed, encounter time, and sensitive waters. The index system suggested by our approach not only dynamically reflects the safety status of the port area, such as meteorology and hydrology, but it also dynamically tracks the navigation of a single ship, reflecting the navigation risk level of a given ship.

**Figure 5:**
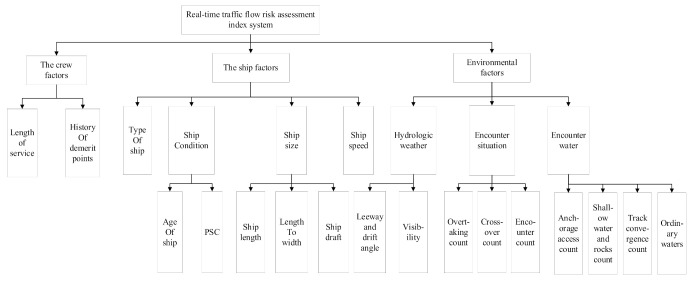
The real-time traffic flow risk assessment index system.

When pursuing efficient safety management, there is a clear need to perform comprehensive real-time monitoring of vessel navigation in port waters. In fact, early warning indicators should be obtained in real time to support real-time monitoring. In existing traffic safety warning index systems, real-time warning indices are most often available, so these cannot support rapid and efficient responses. We propose an index system that eliminates the disadvantages of such indexes, highlights real-time performance, and reflects the natural environment, navigation orders and status, and the location of the ship in sensitive waters. A real-time early warning index system should not only monitor ship navigations in port waters, but should also provide local monitoring of ships, which makes the system more efficient [40,41]. We propose a real-time traffic flow assessment system based on historical marine accident data and expert knowledge derived from a questionnaire-based input, which is summarized in Figure 5.

### 4.1. Appraisal Model

#### 4.1.1. Expert Scoring Method Combined with the Cloud Model

After defining a real-time traffic flow risk assessment index, a crucial step is to determine the weight of each impact index for the risk assessment model. Figure 5 identifies the contents of the parameters of each level of the evaluation system, followed by the corresponding weights associated with the indicators of each level. An expert scoring method is hereafter combined with the cloud model to determine the weight of the different parameters [42].

The principles behind the cloud model were first introduced by Li et al. (1995) based on the fuzzy set theory, statistics, and probability techniques [43]. It has the ability to express ambiguity and randomness in the representation of human knowledge. In past years, the cloud model has been widely used in the fields of imprecise knowledge representation, intelligent control, and system evaluation data mining [44]. A navigation risk assessment of the target water area is a typical multi-level and multi-attribute complex evaluation problem that fits very well with the principles behind the cloud model. It integrates expert evaluations when determining the importance of each index. Given insufficient statistical data and the degree of expert knowledge vagueness, the cloud model can well incorporate the influence of these uncertainties and randomness in the evaluation process [45,46].

Based on expert ratings, we first applied the backward cloud generator algorithm to generate cloud digital features for a certain index. In order to reflect as much as possible the expert evaluation, the forward cloud generator algorithm was then used to generate the expert evaluation cloud map of this indicator. If the cloud droplet distribution of the index weight was relatively scattered, feedback and correction were performed until a more concentrated and stable cloud image was achieved. Further technical details on the specific calculation steps have been widely described in related works [44,46].

#### 4.1.2. Real-Time Traffic Risk Assessment Model

There are many indices affecting the overall navigation risk of the waters as shown in Figure 5. In particular, many ambiguities and uncertainties still remain for each evaluation factor. This led us to evaluate the navigation risks of a given water area based on a fuzzy comprehensive evaluation method. Fuzzy comprehensive evaluation is systematic analysis method that applies fuzzy mathematics principles to analyze things associated with a degree of “fuzziness” [47]. Fuzzy reasoning is a qualitative and quantitative analysis and evaluation method, which combines precision and imprecision. This method has unique advantages when dealing with various complex system problems which are difficult to describe mathematically.

The basic steps of the ship real-time traffic flow risk assessment model based on fuzzy comprehensive evaluation are as follows:

(1)→Determine the respective factor set of the evaluation index.

The evaluation factor set is given by the set of indexes composed of the elements at all levels in the evaluation target, which is set as $\;U=\left\{u_{1},u_{2},\ldots ,u_{m}\right\}$. The evaluation factor set U contains m evaluation indexes, indicating from which aspects the evaluated object can be evaluated, and the evaluation factors can be classified according to their attributes. For example, for the assessment of a ship’s navigation risk, the first level evaluation index should include crew factors, ship factors, and environmental factors, as shown in Table 1.

(2)→Determine the semantic set of the evaluation object.

The semantic set is a set of evaluation grades and is set as $\;V=\left\{v_{1},v_{2},\ldots ,v_{n}\right\}$. It indicates that the evaluation factor set U contains m evaluation indicators, where $V_{j}$ represents the jth evaluation level and n represents the number of evaluation levels. The specific evaluation level can be described according to the evaluation content.

(3)→Determine the weight set of the index.

Let the weight set of the evaluation object be $W=\left\{w_{1},w_{2},\ldots ,w_{m}\right\}$, where $w_{i}\geq 0$, and $\sum_{i=1}^{m}w_{i}=1$; the quantitative expression of the weight value of each factor reflects the importance of each factor. In the method of determining weights, the expert scoring method is combined with the cloud model, which combines fuzziness and randomness to improve the accuracy of qualitative evaluation.

(4)→Evaluation index data processing.

As for the quantitative index, since the quantitative index has different units and dimensions, it is necessary to convert the original index value into the corresponding evaluation value. When the actual value of the quantitative index is larger, the risk assessment value is larger; when the actual value of quantitative index is larger, the risk assessment value is smaller.

As for the qualitative indicators, the evaluation value of the qualitative indicators is determined by the expert scoring method. Several experts in related fields were invited to inform the experts on the evaluation objects and evaluation criteria. The average score of the cloud chart was taken as the evaluation value of the qualitative index.

(5)→Construct a fuzzy relation matrix.

Membership degree is the most basic and important concept of a fuzzy comprehensive evaluation. The so-called membership degree $r_{ij}\;$ refers to the degree that multiple evaluation factors make $v_{j}\;$ an evaluation on $u_{i}$ of an evaluation object. The membership vector is assumed to be $R_{i}=\left(r_{i1},r_{i2},\ldots ,r_{im}\right),i=1,2,\ldots ,n$ and the object from a single index evaluation of the membership of each subset is determined when constructing the membership functions. Some typical membership functions are referred to, such as trapezoidal distribution, made distribution, triangular distribution, and commonly used trapezoidal distribution functions including left trapezoid distribution, right trapezoid distribution and trapezoidal distribution. Formula (7) of the fuzzy relation matrix is obtained by synthesizing the evaluation membership degree of multiple factors.

(7) $$R_{t}=\left[\begin{array}{ccc} r_{t11} & \begin{array}{cc} r_{t12} & \ldots \end{array} & r_{t1m}\\ \begin{array}{c} r_{t21}\\ \ldots \end{array} & \begin{array}{cc} \begin{array}{c} r_{t22}\\ \ldots \end{array} & \begin{array}{c} \ldots \\ \ldots \end{array} \end{array} & \begin{array}{c} r_{t2m}\\ \ldots \end{array}\\ r_{tm1} & \begin{array}{cc} r_{tm1} & \ldots \end{array} & r_{tmm} \end{array}\right]$$

(6)→Fuzzy comprehensive evaluation.

In order to apply a fuzzy comprehensive evaluation, the additive synthesis operator $M\left(\cdot ,+\right)$. When there are many factors sets, the operator can avoid losing information so they can comprehensively consider the influence of all factors and retain all the information of the evaluation results of each single factor, namely, Formula (8):

(8) $$S_{t}=W\cdot R_{t}=(w_{1},w_{2},\ldots ,w_{m})\cdot \left[\begin{array}{ccc} r_{t11} & \begin{array}{cc} r_{t12} & \ldots \end{array} & r_{t1m}\\ \begin{array}{c} r_{t21}\\ \ldots \end{array} & \begin{array}{cc} \begin{array}{c} r_{t22}\\ \ldots \end{array} & \begin{array}{c} \ldots \\ \ldots \end{array} \end{array} & \begin{array}{c} r_{t2m}\\ \ldots \end{array}\\ r_{tm1} & \begin{array}{cc} r_{tm1} & \ldots \end{array} & r_{tmm} \end{array}\right]=(s_{t1},s_{t2},\ldots ,s_{tm})$$

(7)→Real-time risk assessment of a single ship at a given time.

According to the fuzzy comprehensive evaluation method, the ship’s risk in the traffic flow can be obtained. The intermediate value $V=\left\{v_{1},v_{2},\ldots ,v_{n}\right\}\;$ of the evaluation grade is taken, and the weighted average method is adopted to obtain the risk assessment of a single ship’s traffic flow in a certain period of time. $S_{t}$ calculated in Step Six and V here can be used to calculate the risk value of a single ship’s traffic flow in a certain period of time. The formula is as follows:

(9) $$P_{i}(t)=S_{t}\cdot V^{T}$$

(8)→Real-time risk assessment model for ship traffic flow.

Through the calculated risk value of a single ship, the risk assessment of the whole ship traffic flow can be carried out. Because of the maximum risk, the ship has a great influence on the risk degree of the whole ship traffic flow. Therefore, two factors are taken into consideration here: one is the overall risk value of the ship in the period; the other is a consideration of the ship with the maximum risk and the assignment of different weights to it, that is as follows:

(10) $$E=A\cdot \frac{1}{n}\sum_{i=1}^{n}P_{i}(t)+B\cdot \max(P_{i}(t))$$

While A represents the overall risk weight value of the ship, B is the weight value of the vessel at maximum risk. A and B are determined by combining the expert scoring method with the cloud model.

(9)→Risk assessment of a single ship over a total period.

To observe the differences in the risks of each ship in the total time period, it is necessary to carry out the risk assessment for a single ship in the given period:

(11) $$E=A\cdot \frac{1}{n}\sum_{i=1}^{n}P_{i}(t)+B\cdot \max(P_{i}(t))$$

While *t* represents the total period of risk assessment.

### 4.2. Experiments

#### 4.2.1. Real-Time Traffic Flow Risk Evaluation Index

Experts identified for the expert scoring were researchers, captains, and practitioners of the Maritime Safety Administration. Through expert scoring (questionnaires were distributed to experts to rate the importance of the above indicators), the detailed weight rating table of each index was collected and combined with the forward cloud model generator which was used to generate the weight rating cloud map of each index [48,49].

As shown in Figure 5, the risk indicators were divided into three levels, and the survey results of the weight of each indicator at each level were fed back and corrected by the cloud model [50,51]. The risk index evaluation system is a tree structure: the first layer is from the human, ship, and environmental factors, and then from the first layer of the basis of the bifurcation. The relative weights determined by the expert scoring method combined with the cloud model of the port water area real-time traffic flow risk assessment index system are shown in Table 1. 

#### 4.2.2. Real-Time Traffic Flow and Ship Risks in a Port

To apply and evaluate the quantitative index evaluation approach, AIS data of 20 ships in Gulei Port, Zhangzhou, China, on 25 May 2019 were collected by an AIS receiver. In order to value the qualitative evaluation index of the real-time traffic flow risk evaluation system of the Gulei Port, these evaluation values were directly given by experts [52].

To apply the fuzzy comprehensive evaluation method for the risk assessment, a port water real-time traffic flow collection was performed. The index information from a vessel traffic flow of 20 ships was evaluated by combining quantitative and qualitative indices. The ship navigation evaluation grade was valued, and the ship’s navigation risk evaluation in port waters was divided into “low risk”, “lower risk”, “general risk”, “high risk” and “higher risk”, with each grade corresponding to the numerical scoring interval [0, 2], (2, 4], (4, 6], (6, 8). The membership of each basic index was determined by Formulas (12)–(16) to form the membership evaluation matrix.

(12) $$r_{ti1}=\left\{\begin{array}{c} \begin{array}{cc} 1 & x_{ti}<1.5 \end{array}\\ \begin{array}{cc} 2.5-x_{ti} & 1.5<x_{ti}<2.5 \end{array}\\ \begin{array}{cc} 0 & else \end{array} \end{array}\right.$$

(13) $$r_{ti2}=\left\{\begin{array}{c} \begin{array}{cc} x_{ti}-1.5 & 1.5<x_{ti}<2.5 \end{array}\\ \begin{array}{cc} \begin{array}{c} 1\\ 4.5-x_{ti} \end{array} & \begin{array}{c} 2.5<x_{ti}<3.5\\ 3.5<x_{ti}<4.5 \end{array} \end{array}\\ \begin{array}{cc} 0 & else \end{array} \end{array}\right.$$

(14) $$r_{ti3}=\left\{\begin{array}{c} \begin{array}{cc} x_{ti}-3.5 & 3.5<x_{ti}<4.5 \end{array}\\ \begin{array}{cc} \begin{array}{c} 1\\ 6.5-x_{ti} \end{array} & \begin{array}{c} 4.5<x_{ti}<5.5\\ 5.5<x_{ti}<6.5 \end{array} \end{array}\\ \begin{array}{cc} 0 & else \end{array} \end{array}\right.$$

(15) $$r_{ti4}=\left\{\begin{array}{c} \begin{array}{cc} x_{ti}-5.5 & 5.5<x_{ti}<6.5 \end{array}\\ \begin{array}{cc} \begin{array}{c} 1\\ 8.5-x_{ti} \end{array} & \begin{array}{c} 6.5<x_{ti}<7.5\\ 7.5<x_{ti}<8.5 \end{array} \end{array}\\ \begin{array}{cc} 0 & else \end{array} \end{array}\right.$$

(16) $$r_{ti5}=\left\{\begin{array}{c} \begin{array}{cc} 8.5-x_{ti} & 7.5<x_{ti}<8.5 \end{array}\\ \begin{array}{cc} 1 & 8.5<x_{ti} \end{array}\\ \begin{array}{cc} 0 & else \end{array} \end{array}\right.$$

$x_{ti}$—the evaluation value of the *i*th index in the period *t*;

By the algorithm mentioned above, the weights of each index and the fuzzy evaluation matrix were calculated by using the fuzzy operator addition and synthesis operator, and the risk assessment of a ship in a time period was obtained.

(1)→Real-time vessel traffic flow risk assessment.

**Figure 6:**
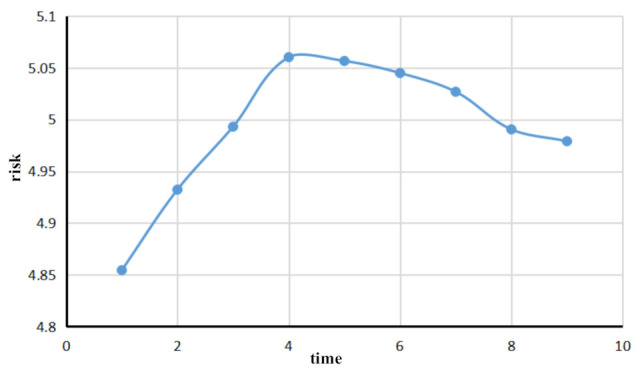
Real-time traffic flow risk variation diagram of port waters.

The traffic flow evaluation for a given port takes into account the whole port traffic flow and the risk in the water area that evolves over time. Based on the historical traffic flow data of a given port, a traffic flow simulation of a section of a given route can be carried out. The main statistics of the ship’s changing parameters encompass ship speed, the number of encounters between ships, and the number of encounters between ships in the given water area. Combined with the existing risk assessment of a single ship in a certain period, Formula (10) was used to consider whether the maximum risk of ships has a greater impact on the overall traffic flow risk, and the weights of parameters A and B, 0.4 and 0.6, respectively, were used to calculate the changes in the overall traffic flow risk. Taking each time point as a node, the overall traffic risk of the target water area was calculated, and the risk-time curve was drawn. The results are shown in Figure 6.

At the initial stage of the ship traffic flow simulation in port waters, as the ship just enters the traffic flow, its speed is low, and the number of encounters between ships is low, and in sensitive waters, the overall risk of traffic flow is low. In the middle stage of the ship traffic flow simulation in port waters, the number of encounters between ships will increase, and the number ships in sensitive waters will increase as well. The overall traffic flow risks will rise. In the late stage of ship traffic flow simulation in port waters, most ships have completed their navigation due to their proximity to the simulated tail section, and the number of ships passing through sensitive waters gradually decreases, so the risk trend of the overall ship traffic flow starts to decline. The appearance of the inflection point in the figure is also caused by the reduction in the number of encounters between ships and the reduction in risk. This conforms to the actual simulation of ship traffic flow in port waters.

(2)→Risk assessment of each ship in port waters over a given period

**Figure 7:**
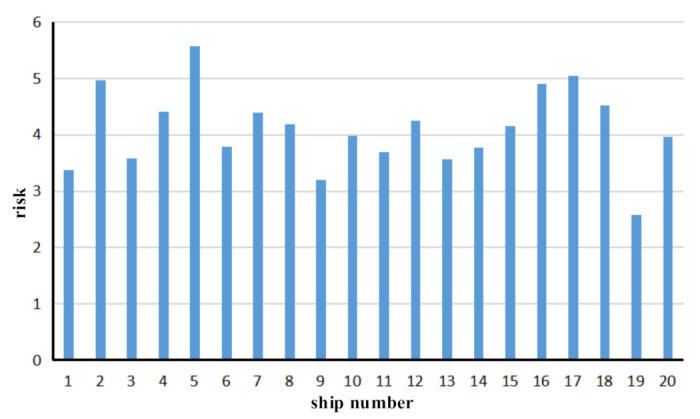
Risk statistical chart of 20 ships in a traffic flow.

For the risk assessment of each ship in port waters for a given period, the risk value of a single ship in the given period can be calculated. Similarly, the risks associated with our sample of 20 ships can be calculated as shown in Figure 7.

The x-axis represents the ship ID, and the y-axis represents the risk value of each ship during the given period. The analysis of the evaluation results shows that the vessel with the highest risk is the vessel with the number five ID, which has the highest length, the lowest draft, and a relatively high-risk level due to the higher occurrence times of the vessel. The ship numbered 19 has a lower speed, fewer encounters, and a lower risk level in the overall voyage. According to the statistical method, the high-risk ships in ship traffic can be identified effectively. This will prompt the relevant departments to make timely preparations to reduce the intensity of maritime supervision work and will reduce maritime risks and accidents.

(3)→Risk assessment of changing ship speed

**Figure 8:**
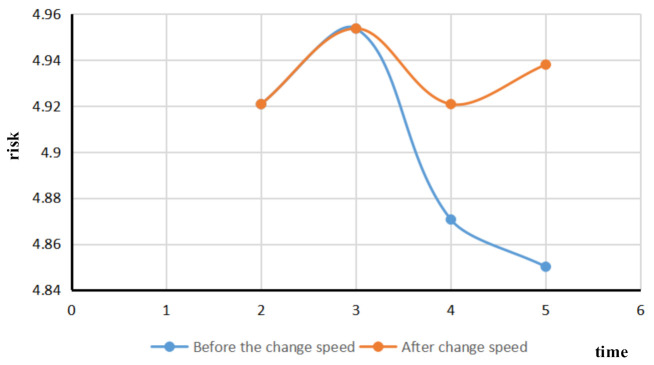
Comparison of changes in ship speed, traffic flow risk.

Based on the real-time risk assessment of existing ships in port waters, the risk variation of ship traffic flow was simulated after a ship changes its speed. The vessel with the MMSI vessel identification number 800000842 was selected to reduce the vessel speed at period four. The comparison diagram of the change of ship speed traffic flow risk is shown in Figure 8.

The blue line represents the variation of ship traffic flow risk before the ship changes speed, with the risk first increasing and then decreasing. The orange line indicates that the risk of ship traffic flow increases after the ship changes speed and then decreases slightly. The orange curve and the blue curve begin to bifurcate at time node three, while the ship of No. 800000842 decreases. Over period four, due to the decrease in ship speed, the ship traffic flow changes, resulting in an increase in the number of encounters between ships in the main channel and the number of encounters between ships in sensitive waters, and a corresponding increase in the risk of ship traffic flow. The risk assessment of a single ship changing its speed in the port area can effectively discover the dynamic risk change of ship traffic flow after the changes of ship speed.

(4)→Risk assessment of visibility changes

**Figure 9:**
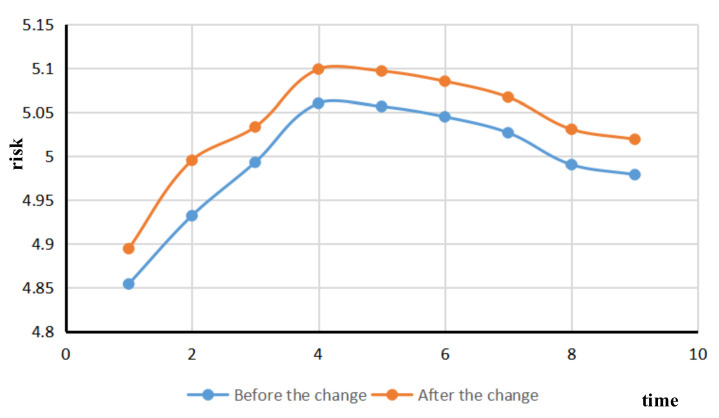
Comparison of traffic flow risk changes with changing visibility.

Based on the existing real-time risk assessment of traffic flow of vessels in port waters, real-time traffic flow risk changes in port waters were observed by reducing visibility. The comparison diagram of real-time traffic flow risk changes in port waters is shown in Figure 9.

To illustrate the analysis of the evaluation results, the blue line represents the change of vessel traffic flow risk before the changes in visibility, with the risk increasing first and then decreasing. The orange line indicates that the overall trend of the vessel traffic flow risk after the changes in visibility is similar to that before the changes in visibility. However, the overall risk of ship traffic flow increases with the decrease in visibility. This is in line with the actual ship traffic flow risk situation. Overall, the real-time risk assessment model of ship traffic flow can effectively evaluate the real-time risk changes of ship traffic flow when the visibility changes.

## 5. Conclusions

This paper introduced a dynamic risk appraisal framework, the objective of which was to address the real-time traffic risk appraisal problem in the fairway. The approach considered static indicators of ship navigation risk based on the simulation of the fairway based on cellular automata and risk indicator statistics. The weight of each risk indicator was determined by combining the cloud model and an expert-based questionnaire. Finally, a fuzzy comprehensive evaluation was used to determine the navigation risk of the channel waters. The proposed approach includes a dynamic traffic flow simulation and a static risk assessment. A dynamic risk assessment of different water areas can be based on the chart to model the channel of the target water area and input the initial traffic flow information. The proposed approach is scalable. Overall, that approach could be used to predict the maritime traffic in real time. The experiments show a satisfactory efficiency and correctness of the whole framework.

Nevertheless, there are still a few model limitations. For example, a CA-based traffic microsimulation model was designed without taking into consideration the vessels outside of the fairway, which will seriously degrade the accuracy of the prediction. Moreover, ship behavior is affected by the traffic and the environment. Some parameters, such as the overtaking possibility, should be obtained from the large data history to improve the accuracy of the prediction. Furthermore, the AIS data mining technology involvement research deserves deeper study, which may benefit the efficiency of the model.

## Figures and Tables

**Table 1:** Relative weight of the real-time traffic flow risk assessment index system of port waters.

Each Sub-Item of the First-Level Index	Sub-Items of Secondary Indicators	Each Sub-Item of the Three-Level Index
The crew factors 0.387	Length of service 0.565	
	History of demerit points 0.435	
Ship factors	Type of ship 0.221	
0.286	Ship Condition 0.265	Age of ship 0.484
		PSC 0.516
	Ship Condition 0.25	Ship length 0.359
		Length to width ratio 0.272
		Ship draft 0.37
	Ship speed 0.265	
Environmental factors	Hydrologic Weather 0.343	leeway and drift angle 0.494
0.328		Visibility 0.506
	Encounter situation 0.269	Overtaking count 0.313
		Crossover count 0.385
		Encounter count 0.302
	Encounter water 0.389	Anchorage access count 0.226
		Shallow water and rocks count 0.298
		Track Convergence count 0.298
		Ordinary waters 0.177

## Data Availability

Not applicable.
